# Comment on “Extraction of Iron from the Rabbit Anterior Chamber with Reverse Iontophoresis”

**DOI:** 10.1155/2015/212095

**Published:** 2015-10-15

**Authors:** Gokhan Ozge, Onder Ayyildiz, Cem Ozgonul, Murat Kucukevcilioglu, Tarkan Mumcuoglu

**Affiliations:** ^1^Department of Ophthalmology, Gulhane Military Medical Academy, Etlik, 06018 Ankara, Turkey; ^2^Department of Ophthalmology, Van Military Hospital, Kayseri, Turkey

We read with great interest the recently published article “Extraction of Iron from the Rabbit Anterior Chamber with Reverse Iontophoresis” by Sun et al. [1]. In this experimental study, researchers aimed to use reverse iontophoresis (RI) to extract iron from the anterior chamber noninvasively. However, we think that there are some points that should be emphasized and reconsidered about this study.

First, as is known, ocular siderosis is caused generally by penetrating eye injury. Intraocular foreign body is most of the time located in vitreous cavity and scleral and/or corneal laceration is seen after penetrating eye injuries. Chiquet et al. reported that, after penetrating eye injury, IOFBs are commonly located in retina (23%), vitreous (48%), sclera (16%), and anterior segment (13%). The common ocular findings upon admission were corneal wound (66%), prolapse or damage of the iris (48%), hyphema (42%), and lens damage (42%) [2]. In this study researchers used an eyecup placed around cornea to perform reverse iontophoresis. We are suspicious about its benefit for penetrating eye injury condition with sutured corneal laceration, corneal edema, and other anterior segment problems.

Our experimental study showed that surgery should be performed within 14 days after penetrating eye injury with iron foreign body [3]; RI might be used for patients without corneal wound as an adjuvant therapy to surgery.
